# Metagenome sequences of unintended bacterial communities from a *Pseudomonas fluorescens* experimental evolution study

**DOI:** 10.1128/mra.00853-26

**Published:** 2026-10-27

**Authors:** Beata Moyo-Heath, Gracious Chigome, Rumbidzai Mushamba, Chimaroke Onyeaghala, Susan F. Bailey

**Affiliations:** 1 Department of Biology, Clarkson University 6704, Potsdam, New York, USA; Michigan State University, East Lansing, Michigan, USA

**Keywords:** experimental evolution, contaminants, *Pseudomonas fluorescens*

## Abstract

We report 16 metagenome sequences from unintended contaminant communities that invaded an evolution experiment initiated with *Pseudomonas fluorescens* SBW25. Low-diversity bacterial assemblies from center and edge locations across eight populations in contrasting uniform or heterogeneous glucose agar plate environments feature four primary genera: *Pseudomonas*, *Chryseobacterium*, *Delftia*, and *Moraxella*.

## ANNOUNCEMENT

Experimental evolution with microbes is a powerful approach for testing evolutionary hypotheses (1). However, despite best efforts, occasionally unintended environmental microbes invade. Contamination is an underreported reality in experimental evolution; however, non-target communities are rarely sequenced or deposited publicly. Characterizing these metagenomes provides empirical data on laboratory contamination profiles and community assembly, potentially revealing novel microbial variants and candidate taxa for future community interaction studies.

We report 16 metagenome sequences obtained from eight experimental populations—four each in two types of environments and sampled at the plate center and edge. Populations were initiated with clonal *Pseudomonas fluorescens* SBW25 and grown at 28°C on M9 salt agar plates (1.5% base agar, 0.4% top agar) with glucose as the sole carbon source provided in “uniform” and “heterogeneous” (1-cm diameter central patch) distributions. Populations were transferred daily via replica stamping (2) for 28 days. Day-28 communities were preserved with glycerol (40% v/v) at −80°C. Unintended contaminant species invaded all replicates between days 21 and 28. We report the Day-28 metagenomic sequences and assemblies as a resource for exploring contaminant invasion dynamics and spatial community structures.

Preserved samples were grown for 20 h in 5 mL Luria–Bertani (LB) media (28°C, 150 rpm) to recover sufficient biomass. DNA was extracted using the DNeasy Blood and Tissue Kit (Qiagen) and sequenced by SeqCenter (Pittsburgh, PA). Sample libraries were prepared using standard SeqCenter protocols with the Illumina DNA Prep Kit (bead-based tagmentation, shearing, and size selection targeting ~280 bp inserts) with IDT 10-bp UDIs. Sequencing was done on an Illumina NovaSeq X Plus platform (2 × 150 bp paired-end reads). Reads were assessed using FastQC v0.11.9 (3) and MultiQC v1.14 (4). Low-quality reads and adapters were removed using fastp (5). All trimmed, high-quality paired-end reads were assembled *de novo* using MEGAHIT v1.2.9 (6) without read subsampling. Assembly metrics were evaluated using QUAST v5.2.0 (7) and reflect all filtered contigs ≥1,000 bp. Taxonomic profiling was performed using Kraken2 v2.1.3 (8) with the standard database and visualized using Krona v2.8 (9). To validate species-level assignments, assemblies were evaluated against reference genomes—*Pseudomonas fluorescens* SBW25 (GCF_000009225.2), *Delftia tsuruhatensis* (GCF_000710165.1), *Moraxella osloensis* (GCF_001013295.1), and *Chryseobacterium indologenes.* (GCF_001013415.1)—using FastANI v0.1.3 (≥95% Average Nucleotide Identity [ANI] cutoff) (10). Default parameters were used for all software, except where otherwise noted.

**Fig 1:**
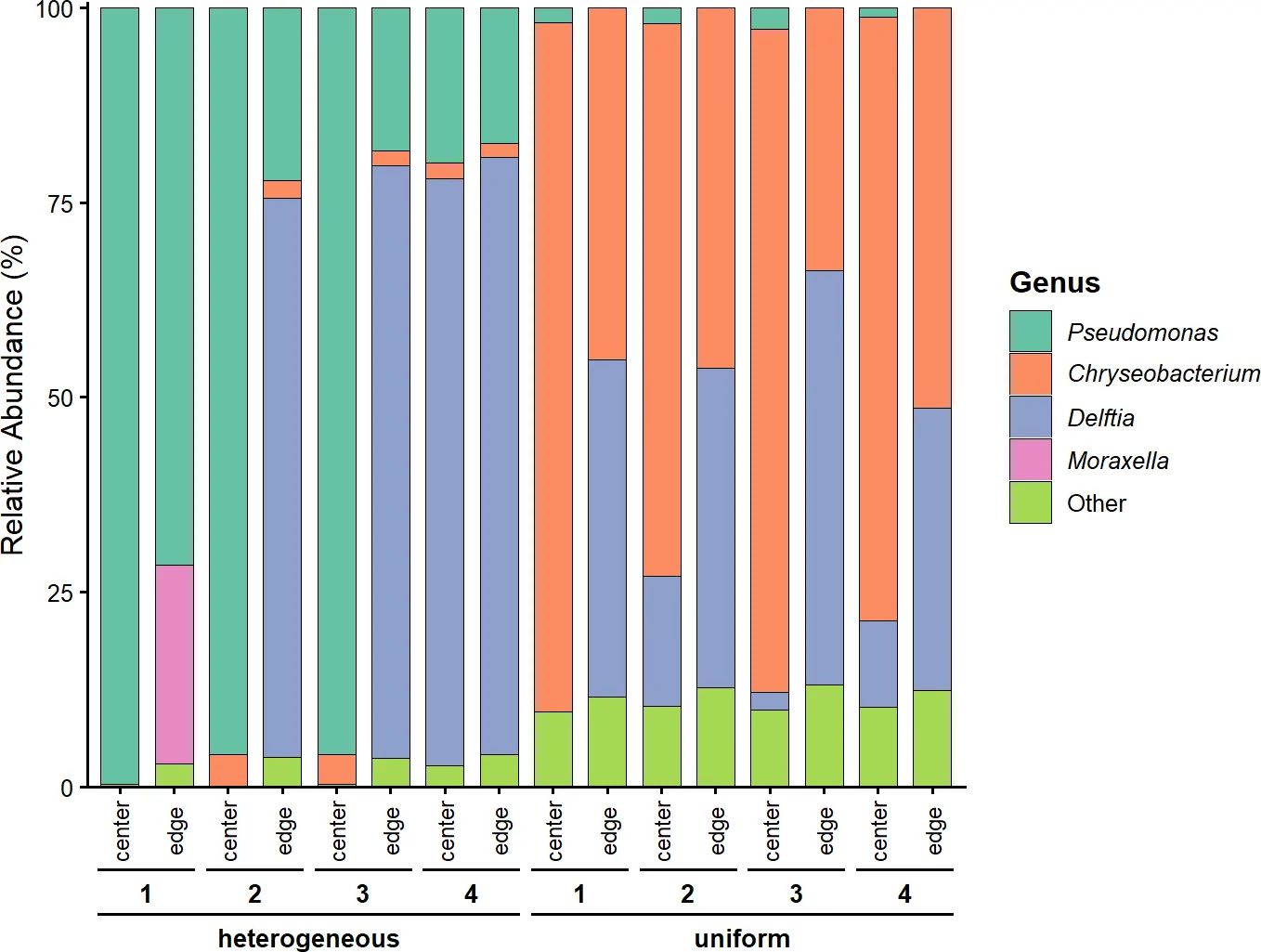
Genus-level community composition of unintended experimental bacterial communities. Abundance profiles are organized by experimental environment type, replicate population, and plate sampling location. 'Other' represents all taxa comprising less than 1% relative abundance.

Sequenced samples produced 6.1–9.7 million reads (150-bp paired-end), with unbinned guanine–cytosine (GC) content of 48.2–60.6%, and unbinned assemblies from 6.7 to 17.6 Mbp (Table 1). Taxonomic profiling inferred low diversity bacterial communities (one to four genera per sample) primarily consisting of: *Pseudomonas*, *Chryseobacterium*, *Delftia*, and *Moraxella* (Fig. 1). Taxa comprising less than 1% relative abundance were designated as “Other.” FastANI confirmed species-level identities for *Pseudomonas fluorescens* (matching ancestral strain SBW25; 98.71–99.97% ANI) and *Delftia tsuruhatensis* (95.37–98.36% ANI). Because *D. tsuruhatensis* typically cannot utilize glucose as a primary carbon source (11), its presence suggests growth via cross-feeding on *P. fluorescens* byproducts, an interaction that future work will explore. Conversely, *Moraxella osloensis* and *Chryseobacterium indologenes* fell below the 95% ANI cutoff across all samples, suggesting these contigs may represent previously unidentified species.

**TABLE 1:** Accession numbers and assembly statistics

BioSample accession	Experimental environment	Sample location	SRA accession	No. of reads	Assembly size (Mbp of contigs ≥ 1 kb)	No. of contigs (≥1 kb)	N50 (bp)	GC (%)	Assembly accession
SAMN54684092	Uniform 1	Center	SRR36861662	8,953,188	8.2	1,860	126,746	48.2	JCDEWF000000000
SAMN54684096	Uniform 1	Edge	SRR36861652	8,029,160	11.7	646	56,944	53.9	JCDEWB000000000
SAMN54684093	Uniform 2	Center	SRR36861661	8,621,376	14.9	2,919	10,594	55.7	JCDEWE000000000
SAMN54684097	Uniform 2	Edge	SRR36861651	6,300,782	11.7	727	49,017	53.8	JCDEWA000000000
SAMN54684094	Uniform 3	Center	SRR36861654	9,688,788	14	3,701	4,034	54.3	JCDEWD000000000
SAMN54684098	Uniform 3	Edge	SRR36861650	6,135,264	11.6	920	37,331	53.7	JCDEVZ000000000
SAMN54684095	Uniform 4	Center	SRR36861653	8,710,538	12.3	1,587	16,848	54.8	JCDEWC000000000
SAMN54684099	Uniform 4	Edge	SRR36861649	6,880,240	11.3	1,202	30,302	53.2	JCDEVY000000000
SAMN54684100	Heterogeneous 1	Center	SRR36861648	6,600,000	6.7	245	44,531	60.6	JCDEVX000000000
SAMN54684104	Heterogeneous 1	Edge	SRR36861658	8,000,000	9.6	361	54,161	55.4	JCDEVT000000000
SAMN54684101	Heterogeneous 2	Center	SRR36861647	7,600,000	11.6	942	24,842	50.4	JCDEVW000000000
SAMN54684105	Heterogeneous 2	Edge	SRR36861657	8,800,000	17.6	1,755	27,997	56.2	JCDEVS000000000
SAMN54684102	Heterogeneous 3	Center	SRR36861660	6,000,000	11.7	934	26,574	50.4	JCDEVV000000000
SAMN54684106	Heterogeneous 3	Edge	SRR36861656	7,400,000	16.5	2,029	31,285	55.3	JCDEVR000000000
SAMN54684103	Heterogeneous 4	Center	SRR36861659	8,600,000	17.3	1,900	27,875	55.4	JCDEVU000000000
SAMN54684107	Heterogeneous 4	Edge	SRR36861655	8,600,000	16.9	2,145	29,030	56	JCDEVQ000000000

## Data Availability

The raw metagenomic sequencing data and assemblies are deposited in NCBI under BioProject accession no. PRJNA1403613. The BioSample accession numbers are SAMN54684092 through SAMN54684107; the SRA accession numbers are SRR36861647 through SRR36861662; and the assembly accession numbers are JCDEVQ000000000 through JCDEWF000000000.
